# 24-Hour Urinary Chemistries and Kidney Stone Risk

**DOI:** 10.1053/j.ajkd.2024.02.010

**Published:** 2024-04-05

**Authors:** Pietro Manuel Ferraro, Eric N. Taylor, Gary C. Curhan

**Affiliations:** Section of Nephrology, Department of Medicine, Università degli Studi di Verona, Verona, Italy (PMF); Channing Division of Network Medicine, Department of Medicine, Brigham and Women’s Hospital, Harvard Medical School, Boston, Massachusetts (ENT, GCC); and Division of Nephrology and Transplantation, Maine Medical Center, Portland, Maine (ENT).

## Abstract

**Rationale & Objective:**

Most previous studies of the relationship between urinary factors and kidney stone risk have either assumed a linear effect of urinary parameters on kidney stone risk or implemented arbitrary thresholds suggesting biologically implausible “all-or-nothing” effects. In addition, little is known about the hierarchy of effects of urinary factors on kidney stone risk. This study evaluated the independent associations between urine chemistries and kidney stone formation and examined their magnitude and shape.

**Study Design:**

Prospective cohort study.

**Setting & Participants:**

We analyzed 9,045 24-hour urine collections from 6,217 participants of the Health Professionals Follow-Up Study and Nurses’ Health Studies I and II.

**Exposure:**

Urine volume and pH, and concentrations of calcium, citrate, oxalate, potassium, magnesium, uric acid, phosphorus, and sodium.

**Outcome:**

Incident symptomatic kidney stones.

**Analytical Approach:**

Multivariable logistic regression analysis incorporating restricted cubic splines to explore potentially nonlinear relationships between urinary factors and the risk of forming a kidney stone. Optimal inflection point analysis was implemented for each factor, and dominance analysis was performed to establish the relative importance of each urinary factor.

**Results:**

Each urinary factor was significantly associated with stone formation except for urine pH. Higher urinary levels of calcium, oxalate, phosphorus, and sodium were associated with a higher risk of stone formation whereas higher urine volume, uric acid, citrate, potassium, and magnesium were associated with a lower risk. The relationships were substantially linear for urine calcium, uric acid, and sodium. By contrast, the magnitudes of the relationships were modestly attenuated at levels above the inflection points for urine oxalate, citrate, volume, phosphorus, potassium, and magnesium. Dominance analysis identified 3 categories of factors’ relative importance: higher (calcium, volume, and citrate), intermediate (oxalate, potassium, and magnesium), and lower (uric acid, phosphorus, and sodium).

**Limitations:**

Predominantly White participants, lack of information on stone composition.

**Conclusions:**

Urine chemistries have complex relationships and differential relative associations with the risk of kidney stone formation.

## PLAIN-LANGUAGE SUMMARY

Kidney stones are common and likely to recur. Certain urinary factors play a role in the development of stones, but their independent roles, relative importance, and shapes of association with stone formation are not well-characterized. We analyzed 24-hour urine collections from individuals with and without kidney stones. Stones were less likely in those with higher urine volume, citrate, potassium, magnesium, and uric acid and were more likely in those with higher calcium, oxalate, phosphorus, and sodium. The acidity of the urine was not related to stones. The urinary parameters showed different degrees of relative importance, with calcium, volume, and citrate being greatest. All parameters exhibited a linear or close-to-linear shape of association with stone formation.

Kidney stones are a common ailment in Western countries, with a high prevalence and tendency to recur.^1,2^ In the pathogenesis of idiopathic calcium nephrolithiasis, urine chemistries have long been known to play a major role.^3^ In particular, because the minerals that make up the most common types of stones are calcium oxalate and calcium phosphate (and mixtures thereof),^4^ urinary excretions of calcium, oxalate, and phosphorus as well as urine volume are expected to directly affect urinary supersaturations for those salts; other urinary constituents such as citrate, uric acid, magnesium, potassium, and sodium as well as urine pH might play a more indirect role.

Most studies of urine chemistries performed in the past have implemented arbitrary thresholds assuming an “all-or-nothing” effect that is not biologically plausible, as reflected by the common occurrence of urinary abnormalities being reported in clinical studies as either present or absent.^5-7^ For instance, the concept of “hypercalciuria,” often defined as urine calcium excretion of 250 mg/24 hours or higher, implies a risk of 0 for an individual with a calcium excretion of 249 mg/24 hours and the same risk for individuals with excretions of 250 and 500 mg/24 hours. On the other hand, assuming a linear effect of urinary parameters on stone risk might be an oversimplification that is not justified by the underlying pathophysiology of urinary saturation, so knowledge of the precise shape of the relationship between each urinary chemistry and stone risk is of crucial theoretical importance and of clinical relevance. No studies so far have taken into account the potential nonlinearity of effects of urine chemistries on the risk of forming kidney stones. Furthermore, to date, no systematic investigation has been conducted to establish a hierarchy of effects of urinary chemistries on stone formation, an endeavor that requires specialized methodological approaches in order to obtain robust results and that would have obvious practical implications allowing clinicians to prioritize interventions to reduce stone recurrence.

Our study analyzed the independent associations between urine chemistries and the likelihood of being a kidney stone former. Specifically, we sought to examine the relationship between each individual urine factor and stone formation, taking into account the role of the other chemistries (as well as of other potential confounders) while also examining the potential for nonlinear relationships, and to establish the relative importance of individual urine factors for stone formation.

## Methods

### Study Populations

The Health Professionals Follow-up Study (HPFS) cohort was started in 1986 with the enrollment of 51,529 male health professionals (dentists, optometrists, osteopaths, pharmacists, podiatrists, and veterinarians) aged 40 to 75 years. The Nurses’ Health Study (NHS) I cohort was started in 1976 with the enrollment of 121,700 female nurses aged 30 to 55 years. The NHS II cohort was started in 1989 with the enrollment of 116,429 female nurses aged 25 to 42 years. For each of the cohorts, the participants completed a detailed baseline questionnaire with information on lifestyle, medical history, and medications. Questionnaires were subsequently mailed every 2 years to update information. This study was approved by the Partners HealthCare institutional review board. Return of completed questionnaires was accepted by the institutional review board as implied informed consent.

### Urine Chemistries

Twenty-four-hour urine samples were collected in 3 cycles. In the first cycle (1994-1999) the participants were eligible if they were ≤65 years old (NHS I) and had no history of cancer or cardiovascular disease. In the second cycle (which started in 2003) the participants were eligible if they were ≤75 years and had no history of cancer (other than non-melanoma skin cancer). In the third cycle (2010-2011) NHS II participants with no history of hypertension were enrolled. Urine samples were analyzed with the system provided by Mission Pharmacal for the first 2 cycles and by Litholink (Labcorp) for the third cycle. Participants with a history of kidney stones were oversampled in the first 2 cycles. Participants with possible over- or undercollections (defined as urinary creatinine excretion in the top or bottom 1% of the non-stone-formers distribution) were removed from the analysis.

### Assessment of Kidney Stones

Participants who reported a kidney stone were sent an additional questionnaire about the date of occurrence and related symptoms. Only symptomatic kidney stones (associated with pain or hematuria) were considered. Self-reported diagnosis was found to be highly reliable by medical record review (confirmed in ≥95% of a sample who completed the supplementary questionnaire).^8^ Stone composition, available for a subgroup of stone formers, was ≥50% calcium oxalate in 77% of NHS I, 79% of NHS II, and 86% of HPFS participants.^8^

### Statistical Analysis

To explore the nonlinear relationships between urinary parameters and kidney stones, restricted cubic splines were created with 4 knots placed at the 5th, 35th, 65th, and 95th percentiles according to Harrell’s criteria.^9^ Logistic regression models adjusted for age, body mass index (BMI), and an indicator variable for cohort and laboratory, with clustered standard errors to account for repeated 24-hour urine collections from the same participant, were used to obtain odds ratios (ORs) and 95% confidence intervals for likelihood of being a stone former across the surface of values of each urinary parameter. All urinary parameters were simultaneously included in regression models to obtain independent estimates of association.

Next, fully adjusted linear spline (piecewise) regression models with kidney stones as the dependent variable were run for each urinary parameter, analyzing 50 equally spaced values and choosing the optimal inflection point based on the highest pseudo-R^2^ value. Finally, nonlinearity was tested by performing a Wald test on the second term of the linear spline. Robustness of the main results was tested by repeating the analyses after removing the 1st and 99th percentiles of each urine parameter to reduce the effect of extreme values.

The dominance analysis was performed using the *domin* program in Stata.^10^ Briefly, dominance analysis allows one to determine the relative importance of several independent variables over an outcome of interest through estimation of the relative contribution of each variable to an overall model fit statistic.^11^ Three tiers of dominance of increasing strength can be established with this technique, including general dominance, conditional dominance, and complete dominance, established through the analysis of the R-square contribution of predictors to a given outcome. General dominance is a simple rank of predictors based on R-square contribution, so if predictor A explains more variance for the outcome of interest of predictor B, and predictor B explains more variance than predictor C, the general dominance rank will be A, B, C. Complete dominance is established between 2 predictors by running all possible subset models; if predictor A outperforms predictor B in all models, then predictor A is said to completely dominate predictor B; for intermediate situations in which predictor A outperforms predictor B on average across model sizes, predictor A is said to conditionally dominate predictor B.^11,12^

A 2-tailed *P* < 0.05 was considered as statistically significant. All analyses were performed using Stata version 16.1 (StataCorp).

## Results

Overall, 9,045 urine collections from 6,217 participants were included in the analysis. A flowchart of the study population is reported in Figure S1, and characteristics of the study population are reported in Table 1. On average, stone formers were older, with a slightly higher BMI, and a larger proportion of men; urinary excretions of calcium, phosphorus, and sodium were higher among stone formers whereas excretions of uric acid, citrate, potassium, and magnesium, as well as volume and pH, were lower.

**Figure 1.**
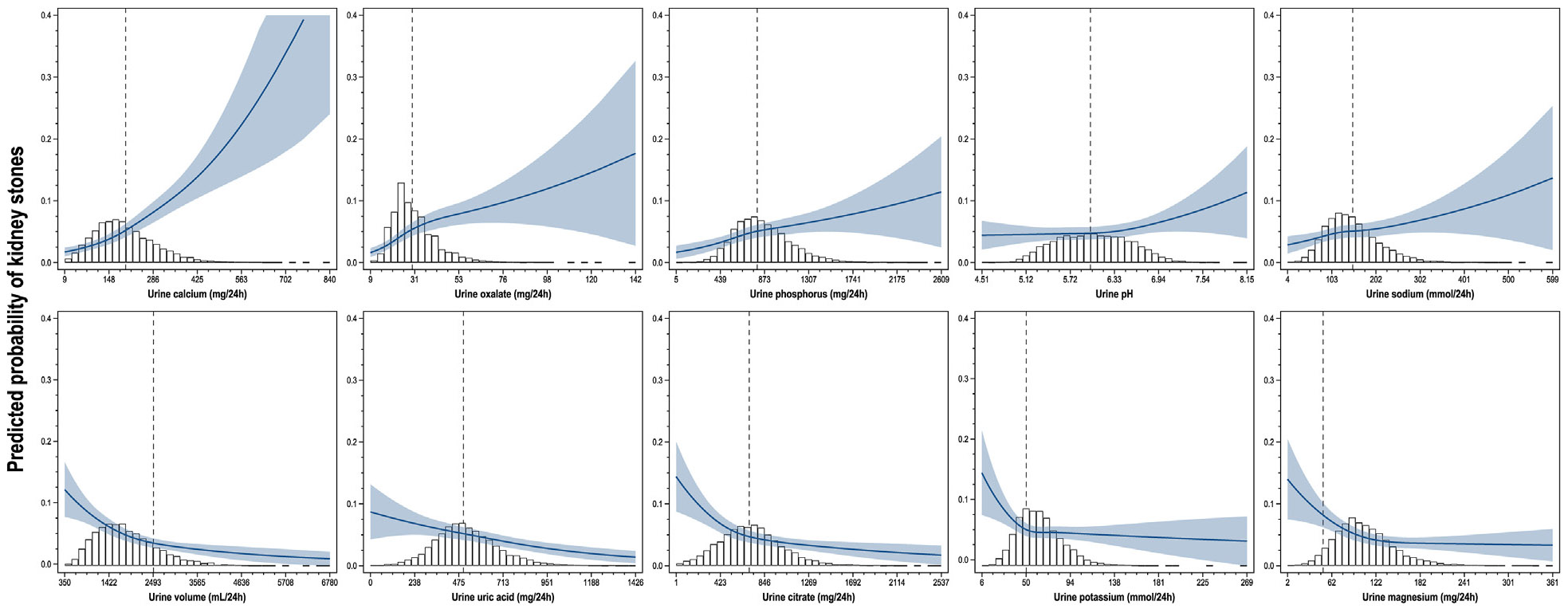
Association between urinary parameters and kidney stones. Urinary parameters were modeled as restricted cubic splines with knots at the 5th, 35th, 65th, and 95th percentiles; estimates were obtained with logistic regression models adjusted for age, body mass index, an indicator variable for cohort and laboratory, and all the other urinary parameters. Dotted lines represent “classic” thresholds for the definition of abnormalities.

The analysis of the association between each urinary factor and stone status is reported in Figure 1 and Table 2. Each urinary factor was significantly associated with stones except for urine pH, which did not reach statistical significance after removal of extreme percentiles (*P* = 0.06). In general, for urinary excretions of calcium, oxalate, phosphorus, and sodium, the association with odds of stone formation was direct, whereas for volume, uric acid, citrate, potassium, and magnesium it was inverse. The analysis of inflection points showed that the relationship was substantially linear for urine calcium, uric acid, and sodium whereas for the other parameters the linear spline model with an inflection point fit statistically better than the linear model. In particular, for urine oxalate, citrate, volume, phosphorus, potassium, and magnesium, the curves flattened after the inflection point; however, the inflection point for volume was located at the extreme of the distribution (with only 2% of all collections greater than that value), so the association could be considered practically linear. The odds ratios provided reflect the change in odds for stones associated with a certain amount of change for a given urine parameter; for instance, for urine calcium (a parameter with no significant threshold) a 50 mg/d increase would result in 31% higher odds; for urine oxalate (a parameter with a significant threshold) a 10 mg/d increase would result in 84% higher odds of stones up to a threshold of 30 mg/d and in 14% higher odds after that threshold. Figure S2 reports the associations expressed as log-odds rather than as odds ratios.

The dominance analysis results are reported in Table 3 and Table S1. When considering general dominance (the lowest tier of statistical dominance), the analysis allowed the identification of 3 broad categories of impact on the outcome, higher (calcium, volume, and citrate), intermediate (oxalate, potassium, and magnesium) and lower (uric acid, phosphorus, and sodium). Conditional and complete dominance allowed bivariate comparisons across urinary parameters: for instance, calcium excretion completely dominated all other parameters except for citrate excretion (conditionally dominated), volume, and potassium excretion (generally dominated) whereas volume completely dominated all parameters except for calcium excretion.

## Discussion

In our study, we performed a comprehensive analysis of the independent role of urine factors and the likelihood of being a kidney stone former. Several of our findings deserve attention. First, we found that all but 1 (pH) of the urinary parameters were significantly associated with stone status. Interestingly, we found a linear, direct association between urine calcium excretion and stone formation. For this parameter, the spline approach showed no apparent inflection point for the “dose–response” curve. That is, there was no definitive threshold for “abnormal” urine calcium. This finding was also suggested by a previous study from our group that showed a linear trend across categories of urine calcium^13^ and was confirmed by the current approach with a larger number of urine collections. These findings strongly suggest that the more the urine calcium can be lowered, the lower the risk of stone formation, which can be used to guide clinical recommendations.

We also found a linear and direct association between urine sodium and kidney stones. Sodium is considered an indirect risk factor for stone formation due to its calciuric effects^14,15^; however, in our study the association was statistically and clinically significant even after adjustment for urine calcium, suggesting a direct effect on crystallization. The inverse association between uric acid and stones was observed in all 3 cohorts and is consistent with a previous study from our group over 10 years ago,^13^ which refuted the belief that higher uricosuria was a risk factor for calcium oxalate stones (“hyperuricosuric calcium oxalate urolithiasis”).^16^ Based on the findings from the current study, uric acid should not be considered to increase the risk of calcium stone formation. Unsurprisingly, urine volume also showed a substantially linear and inverse association with stones; even though the inflection point in the data was statistically significant, it was located at the extreme of the distribution (3.8 L), so the association could be considered linear for practical purposes.

For other factors, we found an inflection point in the dose–response curve from both the visual inspection of the restricted cubic spline analysis and the inflection point analysis. As expected, the associations did not become null after the inflection point but only attenuated in magnitude. Taken together, these findings do not support the notion of urinary abnormalities being defined by arbitrary dichotomous thresholds such as “hypercalciuria” or “hyperoxaluria.”

The lack of statistical association for urine pH is not unexpected; in fact, although we did not have information on stone composition for all study participants, a previous medical record review on a subgroup found that calcium oxalate was the predominant component of kidney stones in this cohort.^8^ Urine pH is not considered a significant factor for calcium oxalate stones. In our study, the restricted cubic spline approach showed a higher proportion of stones at extreme values of high urine pH, likely due to increased probability of calcium phosphate stones.

Our dominance analysis revealed that urine parameters do not have all the same weight with respect to stone formation, with calcium, volume, and citrate being more important. Although no previous study has formally tested the hierarchy of effects of urinary parameters on stone formation, our findings are consistent with the predominant role exerted by calcium excretion and volume over oxalate in calcium oxalate supersaturation reported by Coe et al.^17^ These findings will be important to prioritize the interventions to reduce the risk of stone recurrence. However, the relative importance of urine parameters is likely to vary across different stone types, and our findings likely apply to calcium-based stones, particularly calcium oxalate. Future studies are needed to elucidate this aspect further.

The results of our analysis could be implemented in clinical practice for the management of patients with kidney stones. Dietary and pharmacological approaches to increase urine volume, reduce the urinary excretion of calcium, and increase that of citrate could potentially be prioritized, such as increased intake of fluid and alkali-rich foods, reduced intake of sodium and animal protein, and use of thiazide diuretics and supple/mental alkali. Given the lack of a clear risk threshold for urinary parameters, improving key urinary parameters should be the focus in stone formers regardless of their actual values to minimize the risk of stone recurrence.

Our study has several strengths, including the large sample size, the availability of multiple collections for the same participant (which likely increased the precision of the estimates), and the use of appropriate statistical techniques to address the issues of nonlinearity and relative importance. Our study also has limitations, including predominantly White participants and the lack of information on stone composition for all stone formers. Moreover, several exclusions were applied to the cohorts during the urine collection stages, including cancer, cardiovascular disease, and hypertension; this process might have had an impact on the underlying distribution of certain characteristics such as use of thiazides. However, those are all conditions in which a diagnosis might lead to changes in habits (including dietary habits) as well as to increased rates of imaging tests, which might in turn affect the likelihood of receiving a diagnosis of kidney stones.

In conclusion, several urine parameters are associated with kidney stones, each with an individual dose–response shape and relative strength. Knowledge of these features will help clinicians who manage patients with kidney stones.

## Supplementary Material

Supplementary material **Figure S1:** Flow chart of the study population.
**Figure S2:** Association between urinary parameters and kidney stones expressed as logit.
**Table S1:** Dominance analysis of urinary parameters and kidney stones.

## Figures and Tables

**Table 1.** Characteristics of the Study Population by Stone Status

	Overall	Stone Formers	Non-Stone-Formers
N	6,217	2,577	3,640
No. of collections			
1	3,680 (59%)	907 (35%)	2,773 (76%)
2	2,246 (36%)	1,380 (54%)	866 (24%)
3	291 (5%)	290 (11%)	1 (<1%)
Age, y	57 ± 9	59 ± 11	55 ± 7
BMI, kg/m^2^	26 ± 5	27 ± 5	26 ± 5
Women	5,094 (82%)	1,864 (72%)	3,230 (89%)
Urine parameter			
Creatinine, mg/24 h	1,264 ± 325	1,281 ± 380	1,251 ± 278
Calcium, mg/24 h	200 ± 99	210 ± 107	194 ± 93
Oxalate, mg/24 h	32 ± 12	32 ± 12	31 ± 11
Uric acid, mg/24 h	533 ± 174	517 ± 193	545 ± 157
Citrate, mg/24 h	732 ± 305	668 ± 315	777 ± 289
Volume, mL/24 h	1,890 ± 780	1,660 ± 660	2,050 ± 820
Phosphorus, mg/24 h	860 ± 286	887 ± 313	841 ± 264
Potassium, mmol/24 h	64 ± 22	61 ± 23	66 ± 22
Magnesium, mg/24 h	107 ± 40	105 ± 42	109 ± 39
Sodium, mmol/24 h	149 ± 63	158 ± 66	143 ± 59
pH	6.06 ± 0.51	5.94 ± 0.50	6.14 ± 0.51

Variables are reported as mean ± SD or as count (%); for participants with more than 1 collection, values from the first collection are reported.

**Table 2.** Association Between Urinary Parameters and Kidney Stones

Urinary Parameter (Increment)	N (%) of Collections	Odds Ratio (95% CI)	*P* Value	*P* Value Nonlinearity
Calcium, 50 mg/d	9,045 (100%)	1.31 (1.26-1.37)	<0.001	0.1
Uric acid, 100 mg/d	9,045 (100%)	0.88 (0.84-0.92)	<0.001	0.08
Sodium, 50 mmol/d	9,045 (100%)	1.12 (1.05-1.19)	<0.001	0.3
Volume, 250 mL/d	9,045 (100%)	0.87 (0.85-0.90)	<0.001	<0.001^a^
Oxalate, 10 mg/d				<0.001
<30 mg	4,864 (54%)	1.84 (1.54-2.14)	<0.001	
≥30 mg	4,181 (46%)	1.14 (1.05-1.22)	0.001	
Citrate, 100 mg/d				0.004
<609 mg/24 h	3,362 (37%)	0.83 (0.78-0.88)	<0.001	
≥609 mg/24 h	5,683 (63%)	0.94 (0.90-0.97)	<0.001	
Phosphorus, 100 mg/d				0.04
<630 mg/24 h	1,886 (21%)	1.20 (1.07-1.33)	0.001	
≥630 mg/24 h	7,159 (79%)	1.06 (1.02-1.10)	0.002	
Potassium, 25 mmol/d				<0.001
<53 mmol/24 h	3,159 (35%)	0.55 (0.40-0.69)	<0.001	
≥53 mmol/24 h	5,886 (65%)	0.96 (0.85-1.07)	0.5	
Magnesium, 25 mg/d				<0.001
<138 mg/24 h	7,281 (81%)	0.79 (0.74-0.85)	<0.001	
≥138 mg/24 h	1,764 (19%)	1.01 (0.91-1.10)	0.9	

Estimates obtained with a logistic regression model adjusted for age, BMI, cohort/laboratory and all the other urinary parameters and clustered standard errors to account for repeated collections from the same participant. Estimates refer to a “clinically meaningful” difference in the urinary parameter: calcium, 50 mg/d; uric acid, 100 mg/d; sodium, 50 mmol/d; oxalate, 10 mg/d; citrate, 100 mg/d; volume, 250 mL/d; phosphorus, 100 mg/d; potassium, 25 mmol/d; magnesium, 25 mg/d.

a See text for more details.

**Table 3.** Dominance Analysis of Urinary Parameters and Kidney Stones

Urinary Parameter	Standardized Dominance Statistic	Rank
Calcium	0.270	1
Volume	0.216	2
Citrate	0.137	3
Oxalate	0.099	4
Potassium	0.099	5
Magnesium	0.071	6
Uric acid	0.044	7
Phosphorus	0.037	8
Sodium	0.027	9

The standardized dominance statistic reflects the fraction of variance in the outcome explained by a given urinary parameter (eg, 27% of variance in the kidney stone outcome is explained by urinary calcium excretion).
