# Supplementary material for: 24-Hour Urinary Chemistries and Kidney Stone Risk
Source: Am J Kidney Dis. Author manuscript; Available in PMC 2026 May 13. (PMC13170619; doi:10.1053/j.ajkd.2024.02.010)
Supplement: Supplementary material — Figure S1: Flow chart of the study population. Figure S2: Association between urinary parameters and kidney stones expressed as logit. Table S1: Dominance analysis of urinary parameters and kidney stones. [file NIHMS2168727-supplement-Supplementary_material.pdf]

Figure S1. Flow-chart of the study population

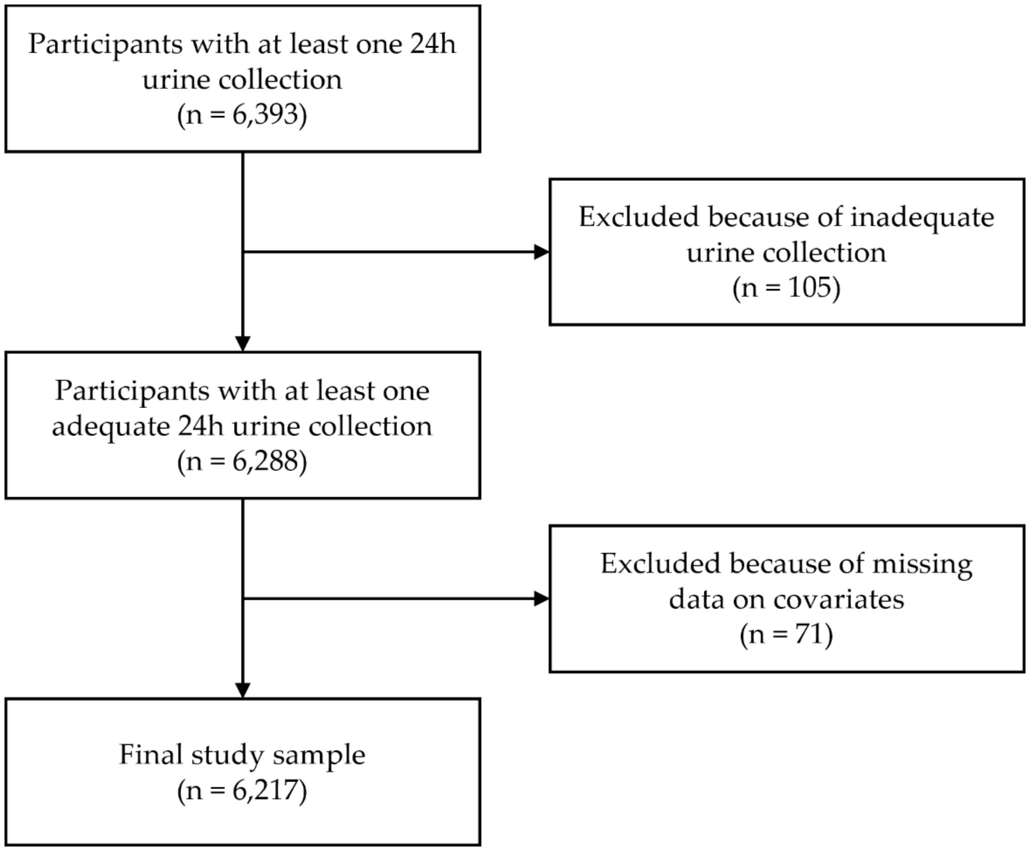

Figure S2. Association between urinary parameters and kidney stones expressed as logit

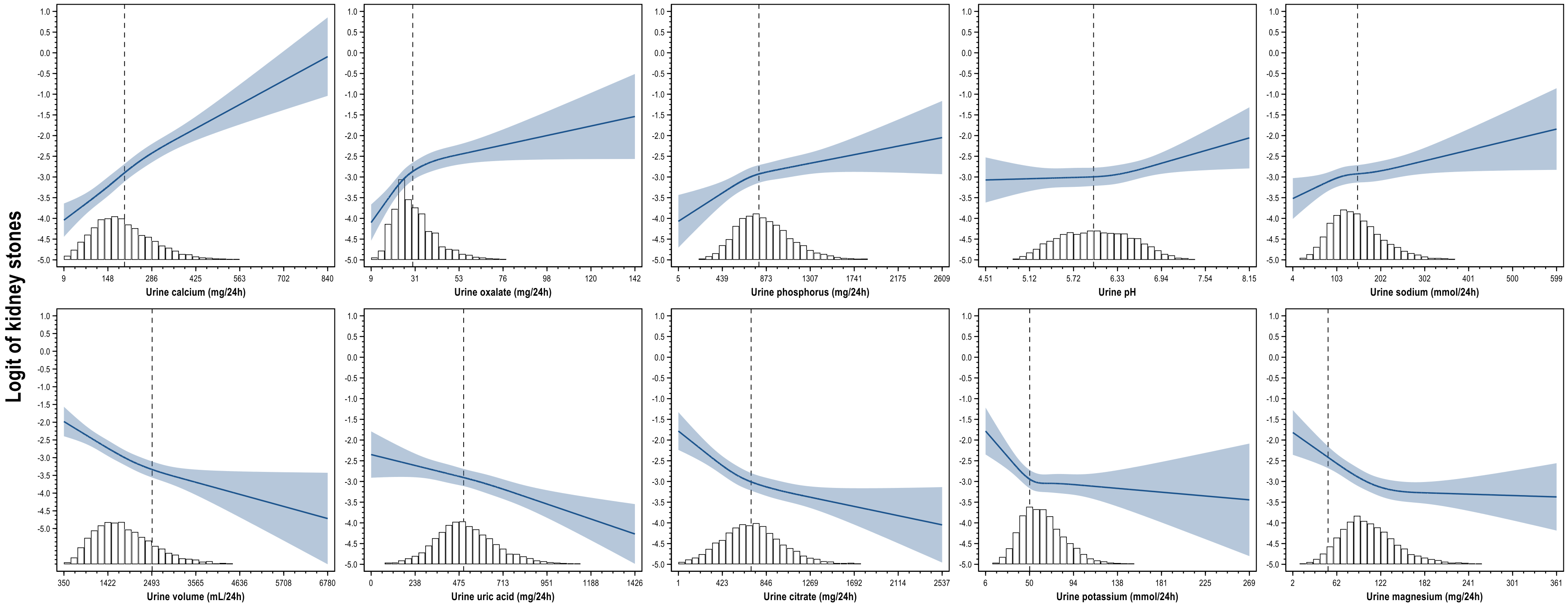

Table S1. Dominance analysis of urinary parameters and kidney stones (strongest dominance designations)

	Calcium	Oxalate	Uric acid	Citrate	Volume	Phosphorus	Potassium	Magnesium	Sodium
**Calcium**	---	***	***	**	*	***	*	***	***
**Oxalate**		---	**			***		*	***
**Uric acid**			---			*			**
**Citrate**		*	***	---		**	*	***	***
**Volume**		***	***	***	---	***	***	***	***
**Phosphorus**						---			**
**Potassium**		*	*			**	---	*	**
**Magnesium**			**			**		---	**
**Sodium**									---

* = general dominance; ** = conditional dominance; *** = complete dominance. Dominating parameters are reported on rows, dominated parameters on columns (e.g., urinary excretion of calcium completely dominates that of oxalate, uric acid, phosphorus, magnesium and sodium; conditionally dominates that of citrate; generally dominates that of volume and potassium)
